# Minocycline Protection of Neomycin Induced Hearing Loss in Gerbils

**DOI:** 10.1155/2015/934158

**Published:** 2015-04-08

**Authors:** Alan M. Robinson, Irena Vujanovic, Claus-Peter Richter

**Affiliations:** ^1^Department of Otolaryngology-Head and Neck Surgery, Feinberg School of Medicine, Northwestern University, Chicago, IL 60611, USA; ^2^Department of Biomedical Engineering, Northwestern University, Evanston, IL 60208, USA; ^3^Department of Communication Sciences and Disorders, The Hugh Knowles Center, Northwestern University, Evanston, IL 60208, USA

## Abstract

This animal study was designed to determine if minocycline ameliorates cochlear damage is caused by intratympanic injection of the ototoxic aminoglycoside antibiotic neomycin. Baseline auditory-evoked brainstem responses were measured in gerbils that received 40 mM intratympanic neomycin either with 0, 1.2, or 1.5 mg/kg intraperitoneal minocycline. Four weeks later auditory-evoked brainstem responses were measured and compared to the baseline measurements. Minocycline treatments of 1.2 mg/kg and 1.5 mg/kg resulted in significantly lower threshold increases compared to 0 mg/kg, indicating protection of hearing loss between 6 kHz and 19 kHz. Cochleae were processed for histology and sectioned to allow quantification of the spiral ganglion neurons and histological evaluation of organ of Corti. Significant reduction of spiral ganglion neuron density was demonstrated in animals that did not receive minocycline, indicating that those receiving minocycline demonstrated enhanced survival of spiral ganglion neurons, enhanced survival of sensory hairs cells and spiral ganglion neurons, and reduced hearing threshold elevation correlates with minocycline treatment demonstrating that neomycin induced hearing loss can be reduced by the simultaneous application of minocycline.

## 1. Introduction

Aminoglycoside antibiotics are effective antibacterial agents that generate ototoxic metabolic products. Systemic administration can lead to destruction of vestibular and cochlear hair cells, causing disequilibrium and/or hearing loss [1–12]. Members of the aminoglycoside family differ in their relative toxicity vestibular and cochlear hair cells, with neomycin and kanamycin A preferentially causing cochlear damage, while streptomycin, gentamicin, amikacin, and tobramycin are preferentially vestibulotoxic [13–15]. Given this toxicity, aminoglycoside treatment for bacterial infections is performed judiciously in developed countries, often being used topically or as the drug of last resort but they are used systemically in underdeveloped countries primarily because they are inexpensive [13].

Ideally their use should be better controlled; however, the addition of an inexpensive agent that ameliorates ototoxicity may be a viable means to prevent unnecessary hearing loss and vestibular disorders. Such an agent would need to suppress the mechanism of ototoxicity. Recent advances have been made in elucidating the mechanism of toxicity to hair cells and have demonstrated substantial apoptosis within twelve hours of exposure to aminoglycoside in guinea pig [16] and initiated within three hours of noise exposure [17]. Knowledge of the mechanistic details of apoptotic induction now provides us with steps in the pathway at which a pharmacologic agent could potentially act to afford inhibition of hair cell death [18–23].

Hair cell apoptosis is believed to be initiated by interference with mitochondrial protein synthesis leading to production of free oxygen radicals and activation of inducible nitric oxide synthetase, producing nitric oxide. Reaction of free oxygen radicals with nitric oxide creates peroxynitrite radicals that initiate the intrinsic or mitochondrial apoptosis pathway [24, 25]. The intrinsic pathway involves proapoptotic proteins Bax and Bak which initiate release of cytochrome c from the mitochondria followed by caspase 9 and caspase 3 activation and cell death [26]. Initiation of the pathway is also influenced by activation of c-Jun N terminal kinase (JNK) and mitogen-activated protein kinase [27]. Theoretically inhibition of these events may ameliorate hair cell apoptosis.

Pharmacologic agents that inhibit hair cell apoptosis would ideally act as early on as possible in the apoptosis pathway and offer long-term protection to maximize their efficacy. Like all pharmacologic agents they would be subject to meeting the administration, distribution, metabolism, elimination (ADME) and toxicology, drug interaction, and specificity requirements required for approval by pharmaceutical agencies worldwide. Finding a new application for an already approved pharmacologic agent to be used for amelioration of hair cell apoptosis would be ideal [28]. Additionally, the apoptotic mechanism of noise-induced hair cell death appears to follow a similar mechanism, so the potential for a pharmacologic agent to treat both causes of hearing loss may be feasible [29].

Minocycline is a long-time worldwide approved inexpensive tetracycline derivative that has antibiotic, anti-inflammatory, free radical scavenging and antiapoptotic induction properties [30–36]. Most pertinent to this study it has been reported to inhibit* in vitro* hair cell death in rat cochlear explants [37]. Therefore, minocycline was tested for its* in vivo* ability to ameliorate hearing loss in gerbils following intratympanic neomycin application.

## 2. Methods

### 2.1. Animals

Six-month-old gerbils (*Meriones unguiculatus*) of either sex (60–80 g) were used for this study and were randomly assigned to minocycline treatment groups. Animal numbers represented were *n* = 6, *n* = 8, and *n* = 7 for 0 mg/kg, 1.2 mg/kg, and 1.5 mg/kg minocycline, respectively. The human equivalent dose for 1.2 mg/kg based on the body surface area normalization method would be 1.2 × (4/37) = 0.13 mg/kg or 7.8 mg in a 60 kg human, assuming *K*_*m*_ gerbil of 4 and *K*_*m*_ human of 37, where *K*_*m*_ is the ratio of body mass to surface area defined in [38]. Based on body mass only the dose would be extrapolated to 1.2 × 60 = 72 mg for a 60 kg human. Additional 3 animals were later included for no minocycline (vehicle alone in ear) and no neomycin controls for histology. Baseline auditory-evoked brainstem responses (ABRs) were measured for the left and right ears of the gerbils.

Following baseline ABR measurements animals received a single intratympanic injection of 40 mM neomycin (Sigma-Aldrich, St. Louis, MO) in Ringer's balanced lactate in the left bulla and Ringer's balanced lactate vehicle alone in the right bulla. 40 mM neomycin was chosen based on it producing only partial hearing loss in our pilot experiments, therefore making it more likely for us to see any minocycline effect. The injection volume was approximately 150 *μ*L, sufficient to fill the middle ear air space. Animals then received either 0 mg/kg, 1.2 mg/kg, or 1.5 mg/kg intraperitoneal (IP) injection of minocycline hydrochloride (Sigma-Aldrich, St. Louis, MO) in normal saline daily, for five days. Day one minocycline injections were given immediately following baseline ABR measurements.

Four weeks after treatment, ABR measurements were performed for left and right ears of each animal. Animals were then anesthetized and sacrificed. Bullae were dissected for histological preparation and analysis of the cochleae. Animal procedures were performed in accordance with the guidelines of the National Institutes of Health and were approved by the Northwestern University Animal Care and Use Committee.

### 2.2. Measuring Cochlear Function with Auditory Brainstem Responses (ABRs)

The method has been described previously [39]. Voltage commands for the stimuli were generated using a computer KPCI 3110-I/O board (Keithley Instruments Inc., Cleveland, OH) inserted into a PC and were used to drive a Beyer DT 770Pro headphone (Beyerdynamic, Heilbronn, Germany). Tone bursts (12 ms duration, including a 1 ms rise/fall) with different carrier frequencies were presented at a rate of 4 Hz. The frequency range was 1.8–50 kHz at three steps per octave. For the experiments, the speculum of the speaker was placed directly in front of the ear canal (quasi free field). The sound pressure was measured with a real head coupler [40].

After the animals were deeply anesthetized, ABRs were obtained by subtracting ipsilateral mastoid potentials from vertex potentials measured relative to a ground electrode, which was placed in the neck. The electrodes were connected to a differential amplifier (ISO 80, WPI, Sarasota, FL). The amplifier setting was at 10,000. Further filtering (300 to 3000 Hz) of the signal was obtained through a Frequency Devices filter (IP90). The sampling rate was 200 kHz, and responses to 100 stimulus presentations were averaged. ABR thresholds were defined as sound levels required for a visible wave II in the response to acoustic stimuli. The noise floor in an averaged ABR recording was typically 1 *μ*V.

### 2.3. Histological Preparation

Bullae were dissected from euthanized gerbils and were fixed for two hours in 4% paraformaldehyde in Phosphate Buffered Saline (PBS, pH 7.4) at room temperature. Postdissection fixation continued overnight at 4°C. Bullae were washed in PBS (pH 7.4) and the inner ear structures were partially exposed by limited removal of surrounding bone. Tissues were decalcified for 48 hours at 4°C in 4% formic acid and 2% sodium citrate, washed in PBS to remove acid and citrate, dehydrated through graded ethanol, cleared in xylene, and embedded in Paraplast-Xtra paraffin wax (McCormick Scientific, St. Louis, MO). Cochleae were sectioned at 10 *μ*m longitudinally to obtain midmodiolar sections mounted on glass slides, dewaxed, hydrated, stained with hematoxylin alone, and dehydrated and cover slips were mounted.

### 2.4. Counting of Spiral Ganglion Neurons

Quantification of spiral ganglion neurons (SGN) was performed in left ears to determine if there was a correlation between minocycline treatment and the respective ABR measurements. Right ear ABRs were used as internal controls to ensure that changes in the left ear ABRs were treatment specific and not systemic or due to the intratympanic injection procedure itself.

The counting was conducted as described previously [39]. In brief, ten consecutive midmodiolar sections were selected to capture images of the spiral ganglion at several different locations along the cochlea. A profile count was performed by two individuals, one blinded to the treatment groups. To do this, every spiral ganglion neuron in a cross section with a nucleus larger than 5 *μ*m and with a nucleolus was counted. The resulting significant cell overcount established by comparison of the stereological count of spiral ganglia sections was corrected for by a factor of 0.71x. The stereologic counting and establishing of the correction factor has been validated and published previously [39].

In addition to counting cells, the cross-sectional area of Rosenthal's canal was determined. Measurements were taken using ImageJ (Wayne Rasband, NIH, public domain software). The program's scale was calibrated and the scale setting was changed from pixels to micrometers. This conversion was accomplished by determining the number of pixels between two lines of the image of a standard slide having 10 *μ*m divisions. Area measurements were obtained by tracing the bony opening of Rosenthal's canal. The total number of pixels within a circumscribed area was calculated and converted into square millimeters.

### 2.5. Histological Evaluation of the Organ of Corti

Hematoxylin stained tissue sections were examined microscopically and digital images captured. The number of observations of hair cells was tallied for the minocycline treatment and corresponding cochlear turns. Ten sections for each cochlear turn were evaluated for each of three animals per minocycline treatment group and no minocycline and no neomycin control group. Cell types scored were inner hair cells (IHC), the three rows of outer hair cells (OHC) 1, 2, and 3 and the three rows of Deiters cells (DC) 1, 2, and 3, and are identified in Figure 3.

### 2.6. Statistical Analysis

**Figure 1:**
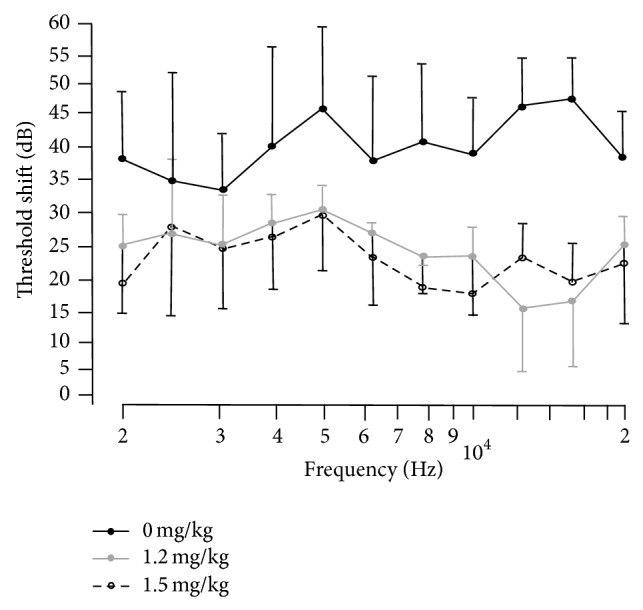
Auditory-evoked brainstem response (ABR) threshold shifts (left ears received transtympanic neomycin) for 0 mg/kg, 1.2 mg/kg, and 1.5 mg/kg I.P. minocycline treated gerbils. Plots represent subtraction of baseline (week 0) thresholds from week 4 thresholds over approximate frequency range of 2–20 kHz. In the 6–19 KHz range the threshold shifts of 1.2 mg/kg and 1.5 mg/kg minocycline treated animals were not significantly different from each other but were significantly less than threshold shifts of the 0 mg/kg animals (see Table 1 and methods for Fisher's exact statistical test of data). Error bars are standard deviation from mean threshold shifts.

ABR thresholds were entered into IgorPro 4 (Wavemetrics) software to generate threshold shift values by subtracting baseline thresholds from week 4 thresholds for each treatment. Threshold shift and frequency curves were plotted with standard deviations (Figure 1). For statistical analysis of the threshold shifts, Fisher's exact test was used to compare minocycline treatments for the 6–19 kHz frequency range, where standard deviations suggested likely significance. Pairwise comparisons were made between 0 mg/kg and 1.2 mg/kg, 0 mg/kg and 1.5 mg/kg, and 1.2 mg/kg and 1.5 mg/kg minocycline. Columns of the 2 × 2 contingency tables consisted of the minocylcline concentration treatment category; rows of the table consisted of the number of animals falling within threshold elevation ranges of either 1–19 dB SPL or 20 dB > 20 dB. The threshold bin range was set as the midpoint between the the 0 mg/kg threshold shift and the minocycline treated threshold shifts (Table 1).

Mean SGN densities were calculated separately for the upper base, middle, and upper middle regions of the cochlear turns for each animal in each treatment group and tested for statistical significance by one-way ANOVA followed by Tukey HSD test using IgorPro 4 (Wavemetrics) software. Qualitative histological examination of the upper base, middle, and upper middle regions of the cochleae was performed on SGN appearance in Rosenthal's canal and the presence or absence of hair cells in the organ of Corti.

## 3. Results

Left ears were treated with neomycin and animals received either 0, 1.2, or 1.5 mg/kg micocycline IP. At week 4 after neomycin left ear ABR thresholds were elevated from their baseline obtained at week 0 for all minocycline treatment groups (Figure 1). To quantify the overall change in ABR threshold, for each treatment group, the average threshold was calculated by averaging the threshold at the different frequencies and by subsequently subtracting the average threshold obtained before neomycin treatment to obtain a threshold shift. Average threshold shifts for the individual minocycline treatments were 40 dB for 0 mg/kg minocycline and 20 dB for both 1.2 mg/kg and 1.5 mg/kg (Figure 1). Threshold shifts for right ear controls were at or close to zero indicating that the ABR threshold elevations in the left ears were treatment specific, not systemic, or due to the intratympanic injection trauma (not shown).

As absolute thresholds beyond the speaker output limit could not be determined in most 0 mg/kg minocycline treated animals only measures below 20 kHz were considered in the calculation of the mean threshold shifts, thereby underestimating the threshold shift in the 0 mg/kg cohort. For example, in some animals, no ABR response could be evoked for stimulus frequencies above 19 kHz, indicating a larger threshold shift than the difference between the sound level for the baseline ABR threshold and the maximum sound level of the speaker. They were deaf at those tested frequencies. Two animals in the 0 mg/kg minocycline group had no detectable thresholds above 12 kHz which meant that they had no quantifiable thershold shift, so for statistical purposes we used Fisher's exact test as described in the methods to include these animals (Table 1). Threshold shifts of 1.2 mg/kg and 1.5 mg/kg minocycline treated animals were not significantly different (Fisher's exact test) from each other but were significantly less than threshold shifts of the 0 mg/kg animals over 6–19 kHz (*P* = 0.028 one sided).

**Figure 2:**
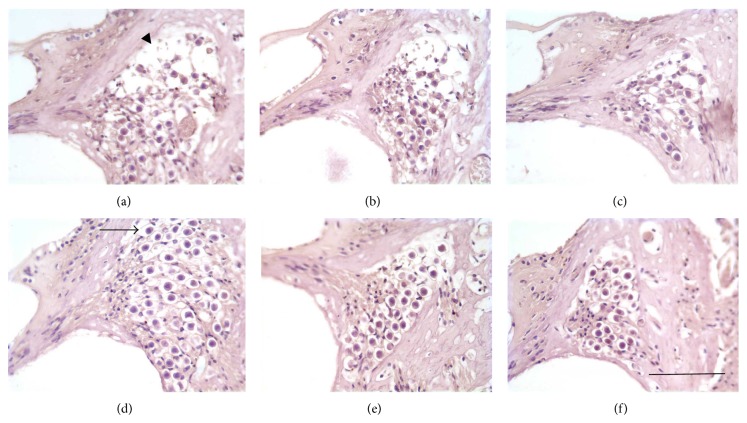
SGN within Rosenthal's canal (arrow head) of the left ear cochlea. Upper middle, middle turn, and basal turn (a, b, and c, resp.) of 0 mg/kg minocycline treatment compared to corresponding turns of 1.2 mg/kg minocycline treated animal (d, e, and f). 0 mg/kg animals show reduced SGN density (arrow head) compared to 1.2 mg/kg. Arrow indicates SGN. Scale bar = 50 *μ*m.

The mean SGN densities in the upper base, middle, and upper middle regions of the left ear cochlear turns are shown in Table 2 and visually in Figure 2. Each animal in each treatment group demonstrated a significant difference by ANOVA between 0 mg/kg and 1.2 mg/kg and 0 mg/kg and 1.5 mg/kg minocycline (*P* < 0.05).

**Figure 3:**
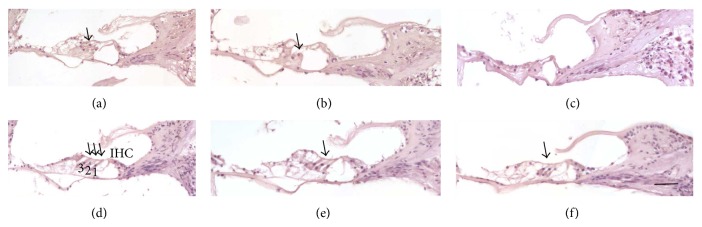
Organ of Corti within the left ears of 0 mg/kg minocycline treated animals (a, b, and c); the upper middle and middle turns (a) and (b), respectively, had identifiable outer hair cells, while upper basal turns were often devoid of hair cells (c). In the corresponding right ear internal animal controls, the upper middle (d) and middle turns (f), had hair cells as the left ears did, while the upper basal turn (f) had outer hair cells, unlike the left ear. This shows that the neomycin treatment of the left ear caused hair cell loss. Panel (d) is annotated to show inner hair cell (IHC) with arrows indicating outer hair cell rows 1, 2, and 3 which lie immediately above the dark purple nuclei of Deiters cells 1, 2, and 3. Solid arrows indicate outer hair cells. Bar = 50 *μ*m.

**Figure 4:**
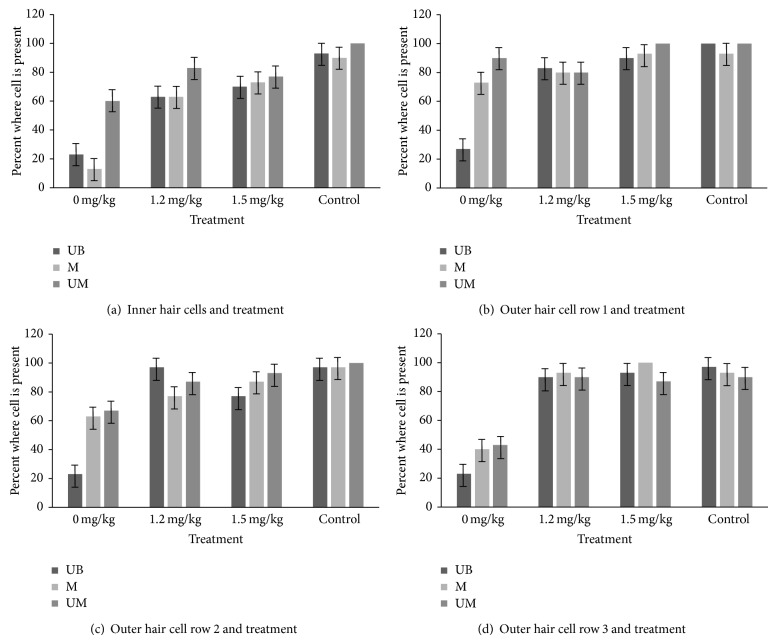
Presence of different cell types was scored in the organ of Corti for each minocycline concentration left ear treatment group in three cochlear turns. Ten sections for each cochlear turn were evaluated for each of three animals per treatment (0 mg/kg. 1.2 mg/kg, 1.5 mg/kg minocycline with neomycin) and no neomycin or minocycline control, such that each turn is represented by a total of 30 sections. The percent of sections where each cell type was present and the corresponding cochlear turns (upper basal UB, middle M, and upper middle UM) are indicated. Error bars represent standard deviation of the proportion of sections where the cell type is present, an absent error bar indicates that all sections contained the cell type. (a) shows percent inner hair cells (IHC) present and (b), (c), and (d) show percent outer hair cell (OHC) rows 1, 2, and 3 present, respectively. Dieters cell rows 1, 2, and 3 showed a similar pattern to the outer hair cells (not shown).

Photomicrographs of organ Corti within left ear cochleae of animals treated with neomycin and those of the corresponding untreated right ear controls demonstrate hair cell toxicity of the neomycin treatment, are shown in Figure 3, and also serve to identify cell types. Cell types within the organ of Corti were scored as ten serial tissue sections of three animals per treatment group and are represented in Figure 4. In the 0 mg/kg minocycline group all cochlear regions had the lowest percent of all cell types evaluated. Of particular note are the hair cells where middle and upper middle turns had the lowest percent decrease in 1.2 mg/kg and 1.5 mg/kg minocycline treatment groups, excluding controls, indicating preservation of hair cells with minocycline treatment. Control animals did not receive neomycin or minocycline and most sections had observable inner and outer hair cells.

## 4. Discussion

We show here* in vivo* that minocycline reduces but does not completely eliminate neomycin induced hearing loss. Furthermore, the specific significant reduction of threshold shift only spans a frequency range of 6–19 KHz, which encompasses part of the upper basal turn extending to the middle turn (Figure 1). However, it is likely that the protected frequency range extends to higher than 19 kHz since the animals not receiving minocycline often had no measurable threshold but even in treated animals these high frequency measures are subject to significant variability due to technical constraints. Our findings are consistent with partial protection of hair cells in rat cochlea explants where maximal prevention of hair cell loss was achieved in explants treated with 100 *μ*M minocycline in combination with the ototoxic aminoglycoside gentamicin [41].

Neomycin is an ototoxic aminoglycoside and cumulative evidence shows that apoptosis of sensory hair cells is the predominant mechanism in ototoxicity, noise, and age-related hearing loss [42]. We, therefore, used the survival of hair cells and SGNs as a metric for minocycline efficacy. The end effect is likely reduced cell apoptosis conferred by minocycline. In general, as to the exact mechanism of minocycline action, no study has been able to absolutely isolate its antiapoptotic and anti-inflammatory properties, which are numerous [43]. With limited understanding [44] of the underlying mechanism(s) of action of minocycline, clinical trials have, and are being conducted to test the efficacy of minocycline in stroke, Alzheimer's disease, multiple sclerosis, spinal cord injury, amyotrophic lateral sclerosis, Huntington's disease, Parkinson's disease, and traumatic brain injury (TBI). The favorable tolerability and safety of minocycline combined with the need for human treatments may justify such wide ranging trials. For example, some combination of the antiapoptotic and anti-inflammatory actions of minocycline may ameliorate traumatic brain injury, with little risk.

We did not address the mechanism(s) of hair cell and SGN protection because our end point was directed at determining a late stage functional hearing benefit, which is a preliminary step in determining efficacy and minocycline dosage. Additional studies within hours of neomycin administration would be required to detect potential antiapoptotic and ant-inflammatory effects of minocycline on hair cells. Current evidence suggests that minocycline inhibition of p38 MAP kinase phosphorylation and inhibition of caspases are possible. On balance, we believe that reporting our results without precise mechanism data is consistent with the sentiment of the public request for publication of even negative minocycline efficacy studies, which may have impeded progress in this field [45].

We did, however, find in pilot studies that there was no difference between wild type and Bax knockout noise induced hearing loss and, therefore, hair cell apoptosis. This lack of protection due to noise exposure in Bax knockout mice is consistent with Bak protein perhaps being the dominant of these two proapoptosis proteins in mitochondrial apoptosis in hair cells. In support of this, others have reported that in age-related hearing loss studies that there was no difference between middle age wild type and middle age Bax knockout mice in their ABR thresholds, while Bak knockout mice had lower thresholds and this correlated with greater density of SGNs and number of outer hair cells only in the Bak knockout mice [46]. Furthermore, several of their other experiments indicated that reactive oxygen species were responsible for apoptosis and that Bak deficiency prevented cell death. Therefore, Bax does not appear to be capable of compensating for lack of Bak expression in age-related hearing loss [46].

Clinically, the use of minocycline for hearing preservation would be limited by its partial efficacy, likely a reflection of redundant apoptosis pathways in hair cells and potential species differences. Several other agents that may partially protect hearing are being examined in animal models of chemical ototoxicity and noise mediated deafness [28]. For example, the known free radical scavenger Allopurinol has been found to provide short term protection from hearing loss in guinea pigs [47, 48]. Combination therapies including minocycline could potentially convey synergistic otoprotection in humans. Also, optimal dosages and routes of administration, including intratympanic injection, have not been investigated. In our model minocycline protection effectiveness had been reached at 1.2 mg/kg. Dosages in other experimental models have demonstrated that efficacy of minocycline in terms of reduced cell death varies widely. For example,* in vitro*, 0.1 *μ*M minocycline has been shown to inhibit the apoptosis promoting enzyme activity of poly (ADP-ribose) polymerase 1 (PARP-1) and confer 85% increased survival of neurons in culture, and 5 mg/kg intraperitoneal minocycline delayed cell death and inhibited caspase-1 and caspase-3 expression in the brains of a mouse model of Huntington's disease [49]. In humans 3–10 mg/kg intravascular doses of minocycline are being evaluated for use in stroke treatment [50]. It should be kept in mind that some applications of minocycline that appear promising in animal models, for example, Huntington's disease [51], may not proceed to human use [52].

Often traumatic brain injury is associated with sound overpressure damage to the cochlea, such as munitions explosions, which can lead to permanent hearing threshold elevation. Such noise induced hearing loss is primarily by apoptosis, and the medical use of minocycline to ameliorate hearing loss could be tested, based on our demonstration of minocycline amelioration of neomycin induced hearing loss. In addition, combination use of minocycline with aminoglycoside antibiotics for bacterial infection could also be evaluated as a means of ameliorating ototoxic hearing loss.

## Figures and Tables

**Table 1:** Shown are the numbers of animals falling within a given ABR threshold shift bin range over 6–19 kHz test range for each minocycline concentration treatment. See Figure 1 representation of threshold shifts for each treatment over the frequency range tested in the ABRs. Fisher's exact 2 × 2 statistical test tables for threshold shift bin ranges for pairwise combination difference tests among 0 mg/kg, 1.2 mg/kg, and 1.5 mg/kg minocycline treatments were performed on animal numbers. Threshold shifts of 1.2 mg/kg and 1.5 mg/kg minocycline treated animals were not significantly different from each other but were significantly less than threshold shifts of the 0 mg/kg animals (*P* = 0.028 one sided).

Threshold shift bin range (dB SPL)	Number of animals (0 mg/kg)	Number of animals (1.2 mg/kg)	Number of animals (1.5 mg/kg)
1–19	0	5	3
20 and >20	6	3	3

**Table 2:** Stereoscopic SGN counts of three animals per treatment group and 30 measures per animal for each of upper basal. Middle and upper middle turns within the cochleae. Statistically significant ANOVA SGN density differences were observed between 0 mg/kg and 1.2 mg/kg minocycline and between 0 mg/kg and 1.5 mg/kg minocycline (*P* < 0.05).

Turn within cochlea	Minocycline concentration		
	0 mg/kg	1.2 mg/kg	1.5 mg/kg
	Spiral ganglion cell density in Rosenthal's canal (cells/mm^2^)		
Upper basal	712 ± 241	1273 ± 374	867 ± 193
Middle	787 ± 306	1201 ± 285	951 ± 315
Upper middle	780 ± 263	1129 ± 188	920 ± 321
Average of all turns	759 ± 263	1203 ± 315	929 ± 276
